# The relationship between Modic changes and intervertebral disc degeneration

**DOI:** 10.1186/s12891-016-1198-1

**Published:** 2016-08-26

**Authors:** Juhani H. Määttä, Alex MacGregor, Jaro Karppinen, Frances M. K. Williams

**Affiliations:** 1 Medical Research Center Oulu, Oulu University Hospital and University of Oulu, Oulu, Finland; 2 Department of Twin Research and Genetic Epidemiology, King’s College London, St Thomas’ Hospital, London, SE1 7EH UK; 3 Finnish Institute of Occupational Health, Oulu, Finland; 4 Faculty of Medicine, Center for Life Course Health Research, University of Oulu, Oulu, Finland

**Keywords:** Body mass index, Disc degeneration, Modic change

## Abstract

**Background:**

Recent reported results have added to the weight of evidence supporting association between disc degeneration and Modic changes. Endplate or Modic changes are also associated with increased body mass index. The most recent study from Teichtahl et al. titled ‘Modic changes in the lumbar spine and their association with body composition, fat distribution and intervertebral disc height – a 3.0 T-MRI study’ showed associations of Modic changes with quantitatively measured reduced disc height and fat mass index. However, there were some facts, which we would like to address in this Correspondence to their article.

**Discussion:**

The different components of intervertebral disc degeneration such as loss of disc height and disc signal intensity have already been shown associated with endplate changes – but not disc height if it is assessed using newer more precise methods of quantitation of disc height. A possible protective effect of different adiposity distribution in the body to Modic change development would be of interest if observed in a longitudinal study in the future.

**Summary:**

Modic changes have been associated with different components of intervertebral disc degeneration such as loss of disc height and disc signal intensity previously. The influence of body fat distribution on endplate changes would be interesting to study longitudinally.

## Background

We were interested to read the recently published study on Modic changes by Teichtahl and Urquhart et al. [1] which used 3 T magnetic resonance imaging of the spine in a small population sample. There is a pressing need to understand better the mechanisms behind the common and costly social problem of low back pain.

We wonder what the rationale for studying fat distribution was when the authors reference a paper showing no evidence of an effect of body mass index (BMI) [2]. In fact, there are several studies published already which have examined endplate changes (i.e. Modic changes). They provide evidence for increasing BMI being associated with endplate changes. Body mass index and waist circumference were both associated with Modic type 2 change among middle-aged male workers in Finland [3]; and among Spanish chronic low back pain patients any Modic change was associated with increasing BMI [4]. Finally, our recent study of a predominantly female twin sample (TwinsUK) found association with increasing BMI and endplate changes [5]. More recently, obesity was proposed to affect to Modic change development through increased spinal forces, i.e. hyperloading, but also may exert influence on adipogenesis, hematopoiesis and osteogenesis [6].

Our work using the TwinsUK registry [5], as well as that of Kerttula et al. [7], examined endplate changes and features of intervertebral disc and found an association with both disc height loss and change in disc signal intensity. While our work lacks T1-weighted images and therefore isn’t strictly as Modic described, the sample is more than ten fold larger than the reported Australian study and likely presents robust findings. Even though disc height loss has been associated with Modic changes, it is true that previous studies have evaluated disc height by semi-quantitative scale as applied by trained personnel using a reference atlas, and not quantitatively. Quantitative measurement is an advantage in the study of Teichtahl et al. [1], but it should be noted that the association of disc height loss and Modic changes have been reported previously.

It would be of interest to determine whether different distributions of adiposity really have opposite effects or whether this reflects chance findings in a cross-sectional snapshot in endplate progression. In TwinsUK data we observed an association between disc height and disc bulge at baseline and an incident endplate change during follow-up of over a decade. Finally, most importantly of all, we showed both intervertebral disc degeneration and endplate changes to be independent predictors of episodes of severe and disabling low back pain.
