# Supplementary material for: The relationship between Modic changes and intervertebral disc degeneration
Source: BMC Musculoskelet Disord. 2016 Aug 26;17(1):371. doi: 10.1186/s12891-016-1198-1 (PMC5002188; doi:10.1186/s12891-016-1198-1)
Supplement: Related texts

# Reply: Response to “The relationship between Modic changes and intervertebral disc degeneration”

**Authors:** Andrew J. Teichtahl, Donna M. Urquhart, Yuanyuan Wang, Anita E. Wluka, Richard O’Sullivan, Graeme Jones, Flavia M. Cicuttini

**Affiliations:** 5 Department of Epidemiology and Preventive Medicine, School of Public Health and Preventive Medicine, Monash University, Alfred Hospital, Melbourne, VIC 3004 Australia; 6 Baker IDI Heart and Diabetes Institute, Commercial Road, Melbourne, VIC 3004 Australia; 7 Healthcare Imaging Services, Epworth Hospital, Richmond, Melbourne, VIC 3121 Australia; 8 Department of Medicine, Central Clinical School, Monash University, Melbourne, VIC 3004 Australia; 9 Menzies Research Institute, Private bag 23, Hobart, TAS 7000 Australia

We thank Määttä et al. for their interest in our work [1]. We agree that a strength of our study is the quantitative measure of intervertebral disc height. This complements the findings from the TwinsUK registry whereby endplate changes (i.e. Modic changes) were associated with semi-quantitative features of intervertebral disc degeneration [5]. We offer intervertebral disc height as a validated, quantitative, easily measured and sensitive alternative to qualitative or semi-quantitative measures of disc degeneration [8].

A major aim of our study was to determine whether body composition was a risk factor for Modic change, with the hypothesis being that adiposity would be a risk factor for such lesions. Previous attempts to examine the association between body mass index (BMI) and Modic change, whether demonstrating significant [3, 4] or non-significant [2] findings, cannot specifically comment on the contribution of adiposity: BMI cannot discriminate adipose or non-adipose mass, nor can it measure the distribution of fat. This is of particular interest since Modic type 2 change histologically represents fat replacement of the vertebral endplate [9].

Additionally, we agree that it would be of interest to further corroborate the differential effect of android and gynoid fat distribution. Nevertheless, our data is compelling: we have shown statistically significant associations (*p* = 0.01) in different biological directions for the exposure of android and gynoid fat. It would seem unlikely that these disparate associations occurred by chance alone, particularly given our modest sample size which would only serve to reduce our chance of showing significant results.

We welcome further research efforts examining these associations, and agree that longitudinal studies would be particularly helpful in determining the predictive nature of not only structural abnormalities, but also symptoms.

## Abbreviation

BMI, body mass index
